# Air Quality Prediction Based on Singular Spectrum Analysis and Artificial Neural Networks

**DOI:** 10.3390/e26121062

**Published:** 2024-12-06

**Authors:** Javier Linkolk López-Gonzales, Rodrigo Salas, Daira Velandia, Paulo Canas Rodrigues

**Affiliations:** 1 Escuela de Posgrado, Universidad Peruana Unión, Lima 15468, Peru; 2 Biomedical Engineering School, Faculty of Engineering, Universidad de Valparaíso, Valparaíso 2362905, Chile; rodrigo.salas@uv.cl; 3 Center of Interdisciplinary Biomedical and Engineering Research for Health—MEDING, Universidad de Valparaíso, Valparaíso 2540064, Chile; 4 Statistical Institute, Faculty of Science, Universidad de Valparaíso, Valparaíso 2362905, Chile; daira.velandia@uv.cl; 5 Center for Atmospheric Studies and Climate Change—CEACC, Universidad de Valparaíso, Valparaíso 2360102, Chile; 6 Department of Statistics, Federal University of Bahia, Salvador 40170-110, Brazil; paulocanas@gmail.com

**Keywords:** air quality, singular spectrum analysis, artificial neural networks, hybrid method

## Abstract

Singular spectrum analysis is a powerful nonparametric technique used to decompose the original time series into a set of components that can be interpreted as trend, seasonal, and noise. For their part, neural networks are a family of information-processing techniques capable of approximating highly nonlinear functions. This study proposes to improve the precision in the prediction of air quality. For this purpose, a hybrid adaptation is considered. It is based on an integration of the singular spectrum analysis and the recurrent neural network long short-term memory; the SSA is applied to the original time series to split signal and noise, which are then predicted separately and added together to obtain the final forecasts. This hybrid method provided better performance when compared with other methods.

## 1. Introduction

### 1.1. Deep Learning Approach in Air Pollution Prediction

Deep learning models have grown substantially due to their excellent performance and flexibility. In particular, the development of the models for the prediction of air quality [1] has become an active research field, highlighting recurrent neural networks (RNNs) and their variations [2] as the tools of modeling with better results in this field. Furthermore, these deep learning methods have been successfully applied in time series modeling to predict air quality [3,4,5,6,7,8,9].

Air pollutants present temporary correlations in their emission and diffusion. Therefore, it is crucial to consider this factor related to the objective variable in quantifying air quality [10]. In this sense, the works of [11,12,13] introduce the temporal correlation of the data to improve the performance of deep learning models. On the other hand, for the prediction of meteorological variables, Pak [10] proposed a convolutional neural network (CNN) and long short-term memory (LSTM) network model, CNN-LSTM, and in the extended LSTM model (LSTME) proposed by [12], the correlations based on PM_2.5_ were captured. A Deep Belief Network (Geoi-DBN) model, with additional variables to PM_2.5_, like temperature, humidity, and wind speed, was presented in [11]. In the deep neural network model proposed by Soh [13], temporal correlation, spatiotemporal, and geographic features were extracted using the LSTM, CNN, and integration of K-nearest neighbors (KNNs) and artificial neural networks (ANNs), respectively; the extracted data were finally assembled into an ANN to produce the prediction value.

### 1.2. Hybrid and Ensemble Methods

Hybrid methods and ensemble methods have been shown to perform significantly better than their counterparts, both in terms of classification and prediction, than single conventional methods such as multilayer perceptron, support vector machines, decision trees, discriminant analysis, and logistic regression, among others [14,15,16]. Moreover, ensemble methods can substantially improve model accuracy; in turn, they are considered supervised learning algorithms, benefiting different training algorithms to achieve higher test accuracy [14,17,18]. For example, Singh [19] applied an ensemble method and principal component analysis (PCA) algorithm to integrate air quality data and forecast air quality index (AQI) values. On the other hand, Xayasouk [20] developed models to predict pollutant concentrations for ten days ahead using the LSTM and a deep autoencoder (DAE). Likewise, Tao [2] proposed a short-term forecasting model that combines 1D convolutional neural networks and bidirectional closed recurrent units (CBGRUs), and Qi [21] proposed a hybrid model integrating graphical convolutional networks and long short-term memory (GC-LSTM) networks to model and forecast the temporal variation of PM_2.5_ concentrations. Recently, Qiao [22] proposed using a self-organized fuzzy neural network to predict the hourly concentration of PM_2.5_ using data from multiple sources.

From the approach of the time series, Hou et al. [23] developed an advanced hybrid wavelet neural network integrated by an autoregressive integrated moving average (ARIMA) model using a fuzzy method, and Díaz-Robles, L.A. et al. [24] proposed a hybrid model that combines ARIMA and an ANN to improve forecast accuracy in meteorological data. Meanwhile, Du et al. [25] developed a hybrid method for estimation and optimization that includes an empirical suite integrated with adaptive filters to remove noise and extract key features from the original data, followed by a wavelet neural network optimized for high estimation accuracy. Also,  Albalawi, F., et al. [26] developed a hybrid HybPAS that integrates the linear regression–deep neural network model and statistics based on signal processing as input values. On the other hand, Siwek, K. and Osowski, S. [27] proposed a system for predicting the PM_10_ concentration in the air using a combination of wavelet transformation and some predictors. They included the least successful predictors, extracted their most important information, and used them in the final forecasting procedure. Likewise, they applied an additional neural network for joint integration. Also, Liu et al. [28], from the bias–variance compensation of the prediction model, introduced the Gradient Boosting Decision Tree in the Bagging training framework to improve the base learning strength and then established the Bagging-GBDT model, using air quality and historical weather data to train the forecast model. In a similar approach, performed in [29], it was concluded that using ensemble ANNs, combined with the multilayer perceptron neural network, leads to better one-step forecast accuracy. On the other hand, Li et al. [30] developed a hybrid QPSO-SVR model to predict the atmospheric concentrations of PM_2.5_ and NO_2_ in the short term, with the QPSO algorithm used to select the optimal parameters that influence SVR performance. Finally, de Mattos Neto, P.S. [31] improved the performance of a hybrid system composed of a genetic algorithm and ANN from the modeling of a residual carried out by an MLP neural network and their hybrid system. This system uses as many series of residuals as necessary to obtain a residual with white noise behavior.

### 1.3. Nonparametric Statistical Methods in Time Series

Statistical methods for time series analysis have been extensively developed and discussed in the literature. However, most of these methods require specialized knowledge to approach their use in correctly modeling data. For example, in the classical context, the series may show a trend. Holt’s exponential smoothing is an option to model linear trend, and without seasonality [32,33], and if, in addition, the series presents seasonality, its modeling is through the smoothing of the Holt–Winters [34,35]. On the other hand, Golyandina [36,37] discussed the singular spectrum analysis (SSA), a nonparametric technique for time series analysis and forecasting, which incorporates elements of classical time series analysis, multivariate statistics, and matrix algebra. Its main objective is to decompose the original time series into a set of components: seasonal, trend, and noise.

Different proposals have been presented in conjunction with machine learning and deep learning algorithms based on this theory. One of the essential issues when analyzing time series is the forecast of new observations. Mahmoudvand [38] proposed a new parsimonious recurrent forecasting model that uses optimal coefficients on the linear combination of the recurrent SSA, and Rodrigues and Kazemi [39,40] developed an improvement in the model fitting performance of the SSA method when the time series is contaminated with outlying observations: a robust SSA (robSSA) approach, where a robust singular value decomposition (SVD) algorithm replaces the standard SVD in the second decomposition step. Furthermore, Rodrigues [41] proposed a method to first smooth out the noise in the tainted data and then calculate the dependency between the variables in the smoothed data. On the other hand, Wang [42] presented a hybrid model that integrates SSA and support machine vector regression (SVR) to forecast failure time series data. Sulandari [43] proposed modeling time series data by obtaining the forecast values with SSA, considering the Linear Recurring Formula (LRF), and then modeling the irregular component by fuzzy systems and neural networks (NNs), and Liu [44] proposed a new hybrid model for short-term prediction based on SSA, a convolutional neural network (CNN), GRU, and SVR, seeing that the SSA decomposes the original series into several components and the CNNGRU predicts the component of trend. In contrast, the SVR predicts the detailed components. Likewise, De [45] proposed the nonlinear combination method, highlighting one for the time series and the other for the residuals, so that both models are combined using a multilayer perceptron (MLP) since it is capable of performing linear and nonlinear combinations. In addition, Samal [46] identified the utility of analytical models to build a system capable of giving a rough estimate of future pollution levels within a considerable confidence interval. Various methods and techniques have been presented to improve the prediction of the concentration of air pollutants.

### 1.4. Comparison with Previous Studies

Traditional models, such as ARIMA and simple variants of neural networks (e.g., MLP), have limitations in capturing nonlinear and stochastic patterns in time series. Compared to studies such as Singh’s [19] that use methods such as PCA and ensemble techniques, this SSA+LSTM proposal goes further by decomposing the series temporally and addressing specific components such as noise, which other studies do not explicitly separate. Recent hybrid methods such as CNN-LSTM [6] or GC-LSTM [21] mainly focus on spatial and temporal integration to improve PM_2.5_ or PM_10_ prediction. This study stands out for the application of SSA as a robust preprocessor that improves signal decomposition and reconstruction before prediction, a strategy less explored in other studies. In works such as Sulandari et al. [43], SSA is used to predict climate variables, but usually with traditional methods (fuzzy systems or SVMs). This study excels by combining SSA with the LSTM, incorporating advanced deep learning techniques to model both linear and nonlinear components.

Likewise, Pak [6] integrates convolutional networks and LSTM neural networks to capture spatiotemporal correlations in the prediction of PM_2.5_. However, it depends on spatially structured data, which can limit its applicability in areas with lower density of monitoring stations. For their part, Qi [21] used graphical convolution networks to model spatial and temporal relationships. This method is effective in regions with a high interaction of pollutants, but its complexity increases with the number of nodes (regions), which can be computationally expensive. Given this, our study presents some alternatives, such as time series preprocessing, since the decomposition of signals into trends, seasonality, and noise improves the predictive capacity by feeding the model with cleaner data. In addition, the versatility without spatial dependence, that is, it does not depend on specific spatial data, makes it more flexible to be applied in regions with fewer resources. Finally, the computational efficiency increases by separating components before using the LSTM, and the computational load is reduced compared with methods such as the GC-LSTM, which process complex data directly. Hou [23] combined ARIMA for linear trends and the MLP for nonlinear ones. However, this method depends on a strict parameterization for ARIMA, which makes it less adaptable. On the other hand, Tao [2] used wavelet-based methods that are effective for decomposing signals, but do not address specific noise modeling as in this research. Thus, our proposal is more flexible, since SSA does not require parametric assumptions, and the LSTM can handle more complex relationships than MLP or wavelet-based methods.

### 1.5. Innovation Points of This Study

This research seeks to consider a new hybrid method that considers the nonlinear interaction of temporal pollution patterns and meteorological variables to improve air quality prediction. This hybrid method was adapted using a nonparametric statistical algorithm and a deep learning approach. This adaptation involves the singular spectrum analysis responsible for separating the trend and seasonality components, i.e., the signal, from the noise components, using the linear recurrent algorithm to forecast the signal. For its part, the long short-term memory neural network will be responsible for addressing the residual part, which is not white noise and presents a stochastic behavior that is not captured in the signal through SSA. In general, the prediction integrates a sum of both what was predicted by the signal component and predicted by the noise component. It is demonstrated how advanced preprocessing methods (SSA) can significantly improve the predictive capacity of neural networks in nonlinear and noisy domains. Accurate air quality prediction is critical in urban contexts to design public policies and risk mitigation strategies. In this sense, this study also proposes the hybrid model as a potential to be implemented in real-time monitoring systems to improve early warning on air pollution.

## 2. Theoretical Foundation

### 2.1. Singular Spectrum Analysis

The singular spectrum analysis algorithm can be written in two complementary stages, decomposition and reconstruction of the univariate time series [36,37], followed by a forecasting stage.

#### 2.1.1. First Stage: Decomposition

**First step: Embedding**. Let $y_{1},\cdots ,y_{N}$ be a time series of length *N*. Considering a window length *L*, the result of this step is a L×K matrix $Y=\left[Y_{1}:\ldots :Y_{K}\right]$, where K=N−L+1 and $Y_{i}={\left(y_{i},\cdots ,y_{i+L-1}\right)}^{T},\;1\leq i\leq K$.
**Second step: Singular value decomposition (SVD)**. In this step, the matrix Y will be decomposed using SVD as $Y=Y_{1}+\cdots +Y_{L}$, where $Y_{i}={\sqrt{\lambda }}_{i}U_{i}{V_{i}}^{T}$, $Y_{i}=0$ when $\lambda_{i}=0$, and $V_{i}=Y^{T}U_{i}/{\sqrt{\lambda }}_{i}$ with $\lambda_{1},\ldots ,\lambda_{L},$ and the eigenvalues of $S=YY^{T}$ and $U_{1},\ldots ,U_{L},$ are the corresponding eigenvectors.

#### 2.1.2. Second Stage: Reconstruction

**Third step: Grouping.** The grouping step corresponds to splitting the elementary matrices into *m* disjunct subsets $I_{1},\cdots ,I_{m}$ and summing the matrices within each group. In our application we will focus on m=2, i.e., only two groups. $I_{1}=\left\{1,\ldots ,r\right\}$ and $I_{2}=\left\{r+1,\ldots ,L\right\}$ which are associated with the signal and noise components, respectively.
**Fourth step: Diagonal averaging.** This step transforms each matrix $Y_{I_{j}}$ into a new series of length *N*. Using diagonal averaging, $Y={\tilde{Y}}_{I_{1}}+\cdots +{\tilde{Y}}_{I_{m}}$, where ${\tilde{Y}}_{I_{j}}$ is the Hankelized form of $Y_{I_{j}}$, j=1,…,m. Considering ${\tilde{y}}_{m,n}^{\left(I_{j}\right)}$ the ${\left(m,n\right)}^{th}$ entry of the estimated matrix ${\tilde{Y}}_{I_{j}}$ and denoting by $\left\{{\tilde{y}}_{j_{1}},\ldots ,{\tilde{y}}_{j_{N}}\right\}$, the reconstructed components in the matrix ${\tilde{Y}}_{I_{j}}$, j=1,…,m, applying diagonal averaging, it follows that
  (1) $${\tilde{y}}_{j_{l}}=\left\{\begin{array}{cc} \frac{1}{j_{\ell }-1}\sum_{n=1}^{j_{\ell }-1}{\tilde{y}}_{n,j_{\ell }-n}^{\left(I_{j}\right)} & 2\leq j_{\ell }\leq L-1,\\ \frac{1}{L}\sum_{n=1}^{L}{\tilde{y}}_{n,j_{\ell }-n}^{\left(I_{j}\right)} & L\leq j_{\ell }\leq K+1,\\ \frac{1}{K+L-j_{\ell }+1}\sum_{n=n-K}^{L}{\tilde{y}}_{n,j_{\ell }-n}^{\left(I_{j}\right)} & K+2\leq j_{\ell }\leq K+L. \end{array}\right.$$

#### 2.1.3. Third Stage: Forecasting

Although there are several versions of SSA forecasting algorithms (e.g., [36,47]), we consider here the recurrent SSA forecasting algorithm, one of the most widely used.

Let $U_{j}^{▽}$ be the vector of the first L−1 components of $U_{j}$, the *j*th eigenvector of $YY^{\prime }$, $\pi_{j}$ denote the last component of $U_{j}$, j=1,⋯,r, and *r* denote the number of eigenvalues used for reconstruction. We can define the coefficient vector $\hat{a}$ as

(2) $$\hat{a}={\left({\hat{a}}_{L-1},\cdots ,{\hat{a}}_{1}\right)}^{\prime }=\frac{1}{1-\nu^{2}}\sum_{j=1}^{r}\pi_{j}U_{j}^{▽},$$

where $\nu^{2}=\sum_{j=1}^{r}\pi_{j}^{2}$. Considering the above notation, the *h* steps ahead out-of-sample recurrent SSA forecasts ${\hat{y}}_{N+1},\cdots ,{\hat{y}}_{N+h}$ can be obtained as

(3) $${\hat{y}}_{t}=\left\{\begin{array}{cc} {\tilde{y}}_{t} & \mathrm{for}\;t=1,\cdots ,N,\\ \sum_{j=1}^{L-1}{\hat{a}}_{j}{\hat{y}}_{t-j} & \mathrm{for}\;t=N+1,\cdots ,N+h, \end{array}\right.,$$

where ${\tilde{y}}_{1},\cdots ,{\tilde{y}}_{N}$, are the fitted values for the reconstructed time series as obtained from Equation (1).

### 2.2. Long Short-Term Memory Recurrent Neural Networks

**Figure 1:**
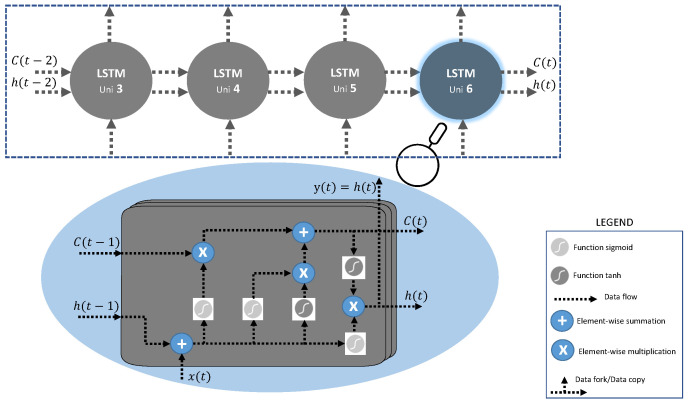
Long short-term memory network. Each neuron has a memory cell and three gates: input, output, and forget. The function of the gates is to safeguard information by stopping or allowing its flow. The input gate determines how much information from the previous layer is stored in the cell. The output gate determines how much the next layer knows about the cell. For its part, the forgetting gate decides whether to keep or forget the information. The LSTM can learn complex sequences.

**Figure 2:**
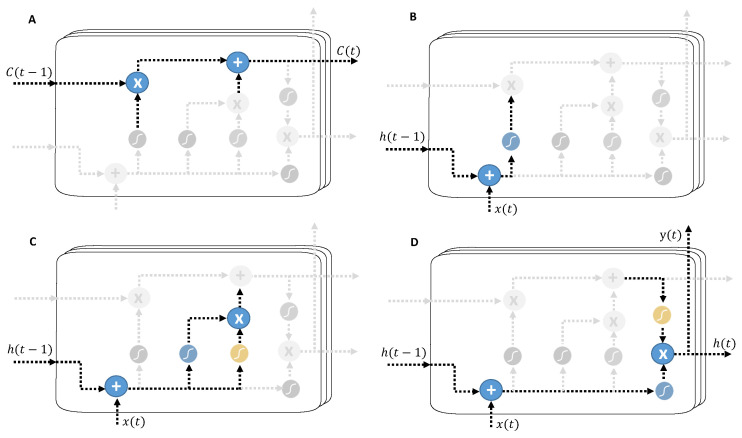
Network long short-term memory detailed. (**A**): cell state; (**B**): forget gate; (**C**): input gate; and (**D**): output gate.

The idea of introducing good loops to produce trajectories in which the gradient can flow for long periods is a fundamental contribution to the initial LSTM model [48]. A crucial addition is that the weight of this loop itself is context-bound rather than fixed [49]. The integration time scale can be changed dynamically by making the weight of this automatic loop closed (controlled by another hidden unit). Even for an LSTM with fixed parameters, the integration time scale may change depending on the input sequence because the model generates the time constants. The LSTM has successfully performed in many applications, such as unrestricted handwriting recognition [50], speech recognition [51,52], handwriting generation by handwriting [53], machine translation [54], image captions [55,56,57], and syntactic analysis [56]. Figure 1 shows a perspective view of the LSTM. They have a link h(t) from one unit to the next, in addition to a cell state, C(t), which is the main flow of information through the LSTM. The cell state is the long-term memory of the model. All that happens afterward is that different filters decide what should be kept or added to the cell state. Cell status is emphasized in Figure 2A. The LSTM also adds and removes information from the cell with the so-called gates, making up the rest of the unit in an LSTM. Gates are a combination of addition, multiplication, and nonlinearities. Nonlinearities are used to “squash” information. For example, the sigmoid function (sigm) is used to “squash” the information to values between 0 and 1, and the hyperbolic tangent (tanh) for values between −1 y 1.

The first gate, so called because it comes from the analogy with logic gates, is the forget gate, emphasized in Figure 2B. The weight of the loop itself (or the associated time constant) is controlled by a forget gate unit $f_{i}^{\left(t\right)}$ (for time step *t* and cell *i*), which sets this weight to a value between 0 and 1 through a sigmoid unit. Intuitively, it controls how much of the raw input and previous hidden state will be remembered. The forget gate in the unit *t* is expressed as follows:

(4) $$f\left(t\right)=\sigma (w_{f}\left(x\left(t\right)+h\left(t-1\right)\right)),$$

where x(t) is the current input vector and h(t) is the currently hidden layer vector containing the outputs of all LSTM cells. Regarding the weights, consider the one that divides $w_{h}$ into several different weights, $w_{f}$, $w_{ff}$, $w_{C}$, and $w_{fff}$. It is worth mentioning that each block must have its own set of weights. All the weight in a complete neural network is trained together with backpropagation, and joint training makes a neural network into a connected whole. The next gate (shown in Figure 2C), called the input gate, precisely decides what to put in the cell state. It comprises another forget gate (denoted ff(t)) but with different weights. It also has an additional module that creates candidates to be added to the cell state. The ff(t) can be thought of as a saving mechanism that controls the amount of input that we will save in the cell state. Then,

(5) $$ff\left(t\right)=\sigma \left(w_{ff}\left(x\left(t\right)+h\left(t-1\right)\right)\right),$$

and

(6) $$i\left(t\right)=ff\left(t\right)\cdot C^{*}\left(t\right).$$

The computation for candidates is $C^{*}\left(t\right)=tanh\left(w_{C}\cdot \left(x\left(t\right)+h\left(t-1\right)\right)\right)$. As seen above, an LSTM unit has three outputs: C(t),y(t), and h(t). With this, the current state of cell C(t) (shown in Figure 2A) can be calculated as follows:

(7) C(t)=f(t)·C(t−1)+i(t).

Let $y\left(t\right)=g_{o}\left(w_{o}\cdot h\left(t\right)\right)$ (where $g_{o}$ is a nonlinear component). For its part, to calculate h(t), a third copy of the forget gate (fff(t)) is needed, which will decide which parts of the inputs and how much to include in h(t) (as a “focus” mechanism that tries to tell which is the most critical part of the cell’s state). We can write fff(t) as

(8) $$fff\left(t\right):=\sigma \left(w_{fff}\left(x\left(t\right)+h\left(t-1\right)\right)\right).$$

Now, the only thing left for a complete output gate (whose result is not o(t) but h(t), emphasized in Figure 2D), is to multiply fff(t) by the current state of the cell squashed between −1 and 1. Then,

(9) h(t):=fff(t)·τ(C(t)),

This allows us to have the complete LSTM network. LSTM networks have been shown to learn long-term dependencies more quickly than simple recursive architectures, first on artificial datasets designed to test the ability to learn long-term dependencies [48,58,59], then on challenging sequence processing tasks where state-of-the-art performance was obtained [51,54].

## 3. SSA+LSTM Hybrid Method

**Figure 3:**
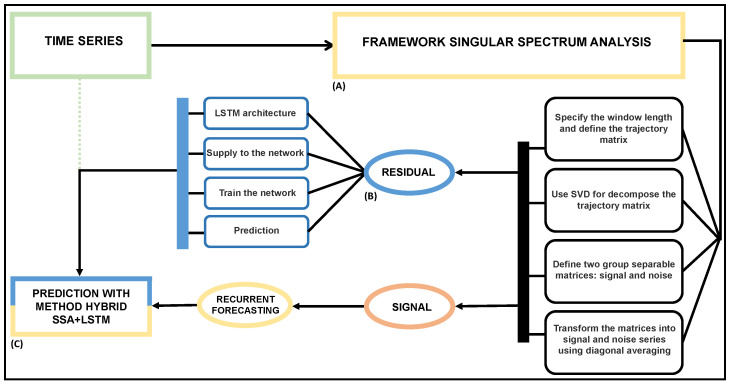
A time series in (**A**). It is executed according to the singular spectrum analysis mechanism (**B**). The residuals are captured and treated according to the characteristics of the long short-term memory network. Finally in (**C**), the prediction is made by adopting properties of both SSA and the LSTM.

The architecture of the SSA+LSTM method is represented in Figure 3. Detailed descriptions are given below.

**Figure 4:**
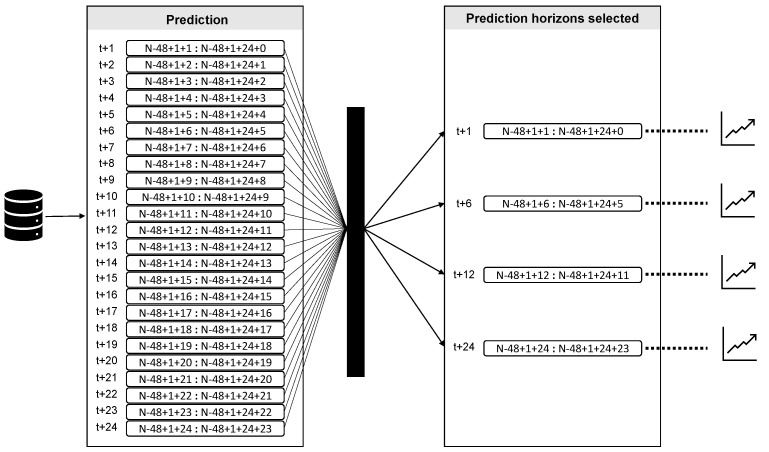
Prediction horizons. Data were normalized within the interval [0, 1]. Then, they were divided into two matrices: training and test. The models were trained with N−48+1 data and tested with the last 48 data. For the time series, 10 simulations were carried out with the adapted approach (singular spectrum analysis and long short-term memory), and the model with the best performance was selected. The results for all models correspond to the prediction of one, six, twelve, and twenty-four steps ahead of the test set. Likewise, Table 1 shows the three metrics that evaluated the performance of the adapted methodology.

- **Sequence 1.** Model and forecast the time series using the SSA algorithm.
  1. Set the length of the window and find the trajectory matrix.
  2. Decompose the trajectory matrix using SVD.
  3. Define the two groups of separable matrices, both signal and noise.
  4. Reconstruct each matrix in a time series using the diagonal averaging algorithm.
  5. Use the recurrent forecasting algorithm to compute forecast values.
- **Sequence 2.** Model the residuals with the LSTM neural network.
  1. Get the residual generated in the above sequence (SSA-RF).
  2. Set the neural network architecture to determine the inputs, nodes, and activation functions used in the hidden layer and the output. For univariate time series forecasting, one output unit is considered.
  3. Obtain the weights that connect each input node with each hidden node, and those that connect each hidden node with each output node using the backpropagation algorithm through time, the same one that allows the solving of two possible consequences related to the gradient, the tendency to infinity when the value is high, or degradation when the value is too small, due to recurring connections.
  4. Calculate the predicted values for the residual generated by the SSA-RF using the LSTM neural network.
- **Sequence 3.** Forecast the time series with the hybrid method SSA+LSTM.
  1. Compute the final values through the sum between the predicted values obtained by the SSA-RF and the predicted residuals obtained by the LSTM network.
  2. Compare the RMSEs, MAEs, and MAPEs obtained from the predictive method (see Figure 4 for details).

### Parameter Settings and Experimental Software Platform

In this study, different functions from both R Studio (2024.09.1+394) and Python (3.11.10) were used. For data imputation, the mice R package’s mice function was used. Also, the MinMaxScaler function from sklearn.preprocessing was used to scale the data. Subsequently, the pytorch, typical of Python, was used to support all the machine learning algorithms. The ssa function of the Rssa R package was also used to model the signal after signal and noise decomposition. On the other hand, the model parameters of the ARIMA model were estimated with the auto.arima function of the R package forecast. In this scenario, the parameters configured for the LSTM and MLP are detailed as follows: hidden.layer.size=10, dropout.prob=0.1, epochs equal to 150 and optimizer=torch.optim.Adam.

## 4. Results and Discussion

**Figure 5:**
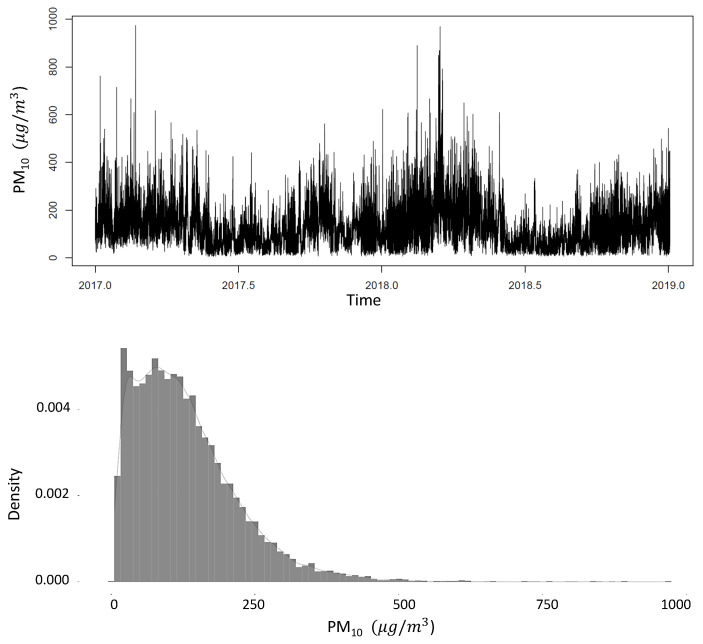
Time series air pollution and the description of the behavior of the contaminant through the histogram.

This section presents the statistical analysis on the original time series of PM_10_ concentration (see Figure 5). Likewise, the evaluation of the models is included. The dataset comprises two years (from 1 January 2017 to 31 December 2018) and includes PM_10_ (μg/m^3^) concentrations for the year, month, day, and hour. These data were obtained from the Servicio Nacional de Meteorología e Hidrología website of Peru, which provides air quality data for the entire country.

**Figure 6:**
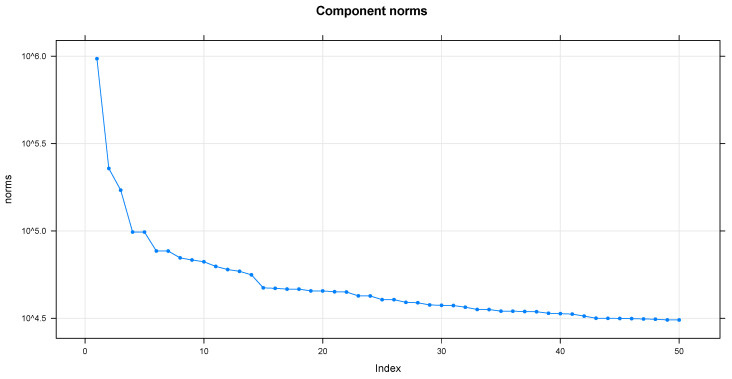
Eigenvalues—SSA.

Figure 6 presents the singular values or eigenvalues of the matrix *X*. The singular values were obtained through the singular value decomposition in step two of the decomposition stage, and their behaviors can be observed through the spectrum of the singular values of the trajectory matrix. One of the essential characteristics to observe is that the first singular value, the one with the highest absolute value, corresponds to the trend component of the series. Other helpful information is provided by checking for breaks in the singular value spectrum. In general terms, a purely residual noise series produces a slowly decreasing sequence of singular values. Since a slight and continuous decrease of the singular values is visualized, it can characterize the beginning of the noise of the time series. It is a way to validate the importance of each component. The main idea is to choose components that allow for better separability between signal and noise. In this case, we consider the first 14 components for the signal and the remainder for the noise.

The SSA calibration depends on two parameters: the window length *L*, and the number of eigentriples used for reconstruction *r*. The choice of improper values for the parameters *L* or *r* yields incomplete reconstruction, and the forecasting results might be misleading [38,60]. Although the choice of these parameters is of crucial importance, no theoretical solution has been proposed for their choosing. An overall agreeable suggestion to choose the window length is that it be close to the middle of the time series and proportional to the number of observations per period (e.g., to 12 for monthly time series). However, this choice does not guarantee the best predictions [38,60] for making a parameter choice according to the available data and intended analysis.

**Figure 7:**
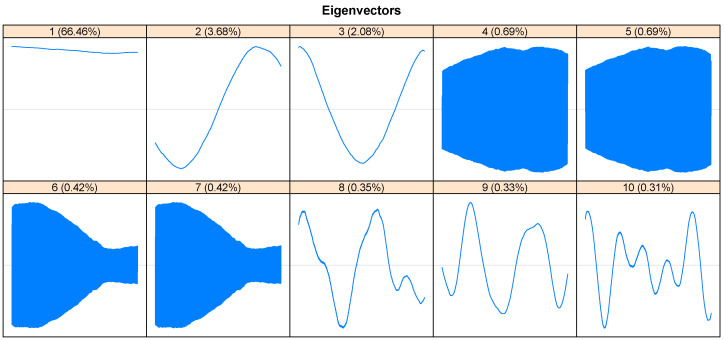
Eigenvectors—SSA. The first 7 components have useful information and the remaining components can be considered noise.

**Figure 8:**
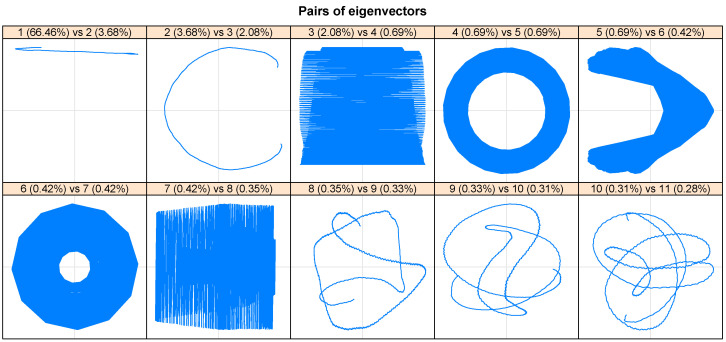
Pairs of eigenvectors—SSA. It is mainly observed that with the pair 6 (0.42%) vs. 7 (0.42%), the size of the seasonality is predicted since it can be differentiated that there is a seasonality of 12 by the number of sides reported by the figure. This is summarized here, in that the pairs 4 (0.69%) vs. 7 (0.69%) and 6 (0.42%) vs. 7 (0.42%) are components that are related to each other; that is, they are close to each other.

Note that the first singular value (eigenvalue of most significant magnitude, rank 1) corresponds to the trend component of the series. Once this trend component has been identified, the paired periodic components are determined. In this way, in the spectrum of the singular values (Figure 6), the periodic paired components composed, mainly by 4–5 and 6–7, are observed, which constitute the seasonality pattern of the original series. From rank 15 onwards, a slight and continuous decrease of the singular values is displayed. Also, there is a break in Figure 6 (component 14); this establishes the boundary between signal and noise. In this context, Figure 7 complements what is observed in Figure 6, since it demonstrates the similarity of components 4, 5, and 6, 7 are also complemented by Figure 8 (seasonality, since the phenomena that occur during a time are repeated in each identical time frame; that is, phenomena that occur daily at a particular time, because the scale is hourly). On the contrary, the rest of the reports are considered trends.

**Figure 9:**
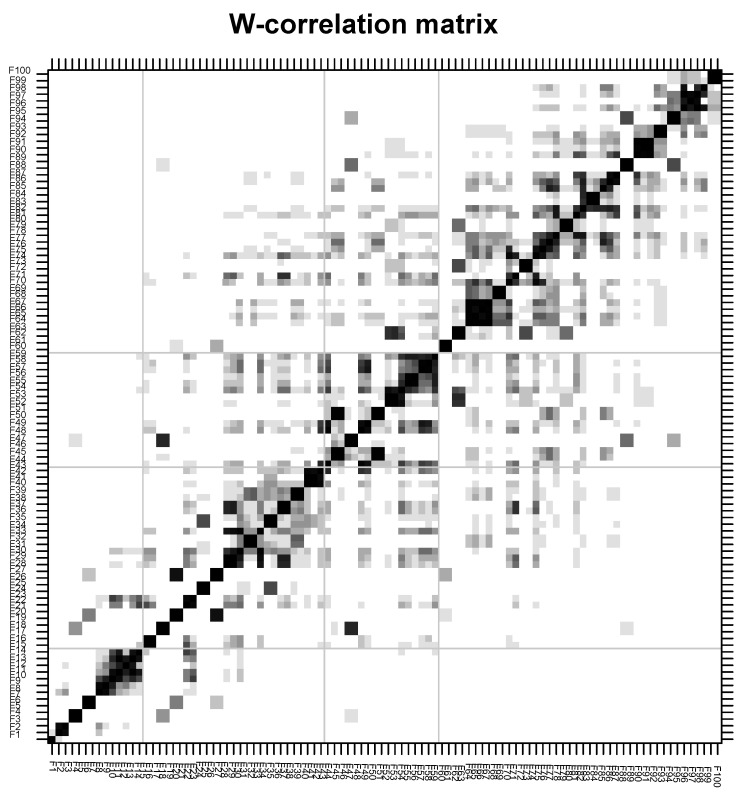
Correlation matrix—SSA. As observed, the diagonal indicates the autocorrelation of each component; in this case, there are 100 components. For example, the black squares represent a w-correlation of 1. Focusing on components 4, 5, 6, and 7, a high correlation is observed, the same as was observed in previous reports. They were similar in terms of magnitude and behavior. This similarity means that they can be grouped together. This report aims to maximize the correlations within the signal and within the noise, and minimize the correlations between the signal and the noise.

For its part, Figure 9 reports the weighted correlation derived from the concept of separability. This is linked to how to choose parameters to build a suitable decomposition of the time series. Furthermore, it characterizes how well the different components can be separated from each other. Finally, it is worth mentioning that the decomposition of the time series will only be successful if the resulting additive components of the series are separable from each other. In this way, the singular value decomposition and eigentriple grouping steps rely on this property called separability. According to [36], in practice, there is no exact separability, only approximate ones. Typically, separability quality is assessed through a natural measure of dependency between the substrings. This measure is called weighted correlation or W–correlation (Figure 9). This plot can be used to check the quality of separability. In turn, it is considered a grouping technique of the components resulting from SVD. What the SSA seeks is to decompose the original series into a sum of series that allows each component to be identified. These components can be observed in Figure 9. This report is based on separability, which allows the choosing of parameters to build an adequate decomposition of the series. SVD and eigentriple grouping rely on this concept [36,61].

**Figure 10:**
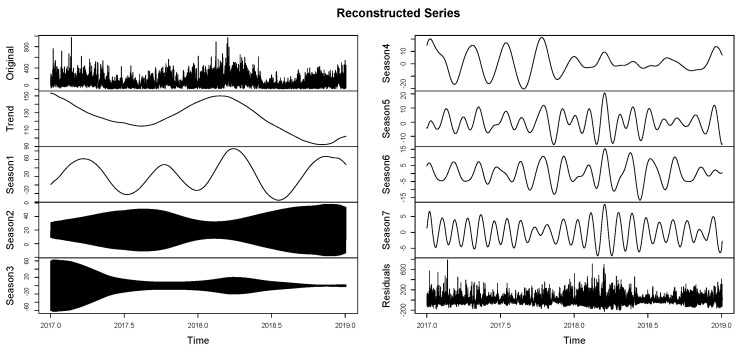
Reconstructed series—SSA. Extraction of information from the time series: trend, seasonality, and residuals.

**Figure 11:**
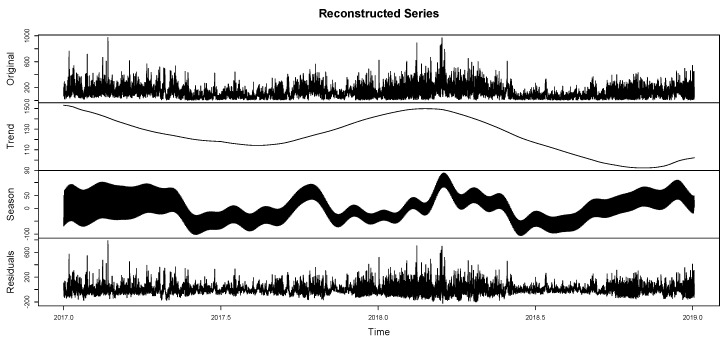
Reconstructed series II—SSA. Clusters of the different seasonalities.

**Figure 12:**
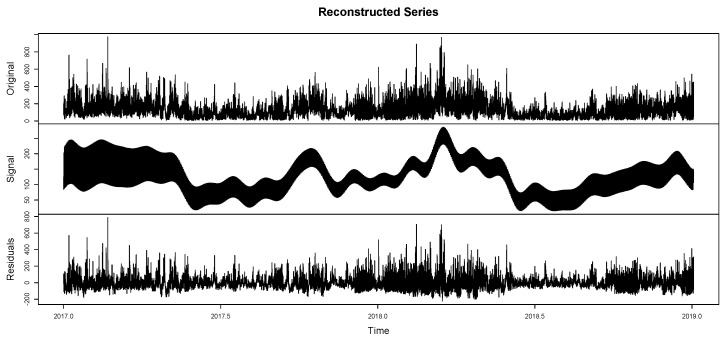
Reconstructed series III—SSA. Here, the original series, the signal, which describes its behavior based on the composition of both trend and seasonality, and finally, the noise (residuals), are integrated.

**Figure 13:**
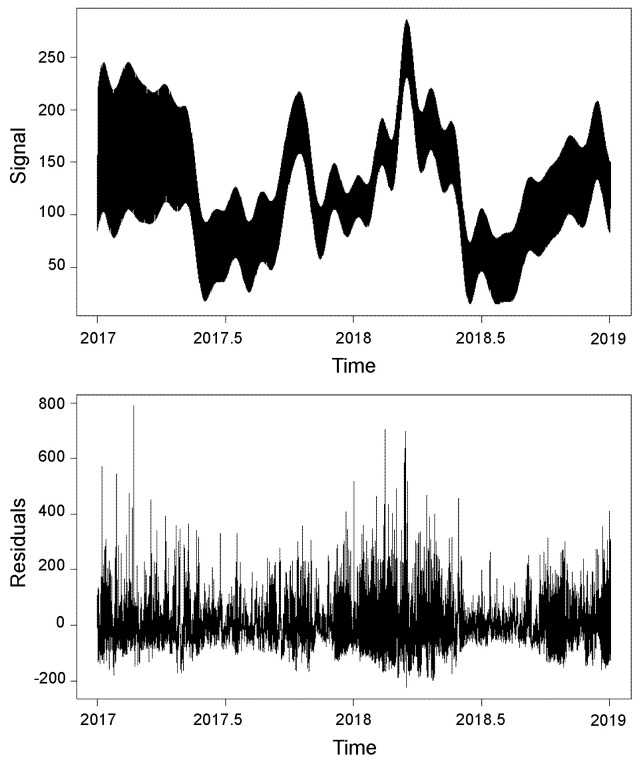
Signal and noise components after the SSA decomposition. The signal includes the deterministic part of the series. The noise includes the stochastic unstructured residuals of the series, which is not white noise.

The components of the series can be differentiated as trend, seasonality, and residuals. This allows for evaluating the grouping of seasonalities (Figure 10) according to the previously reported components. On the other hand, in Figure 11, it is observed that this report clusters the different seasonalities seen in Figure 10. The same components of the time series are observed in a classified manner. This is because the seasonalities can be grouped by similarity, only defining the original series, the trend, seasonality, and residuals. Figure 12 shows the integration of the original time series and the two fundamental components of this process: signal and residuals. The signal will obviously be used within the recurrent forecasting, and the noise will enter the LSTM (both observed in Figure 13). In this context, it is emphasized that the reported noise is not white noise. It is a stochastic behavior not captured in the signal using singular spectrum analysis. The critical episodes in the time series become complex components for the SSA to capture.

### 4.1. Accuracy Measures

In order to evaluate the predictive performance of the different air pollution concentration prediction methods, three performance metrics were used in the study, which are described in Equations (10)–(12). The Mean Absolute Percentage Error (MAPE) and the Mean Absolute Error (MAE) can be used to measure the distance between the forecast data and the actual data; the Root Mean Squared Error (RMSE) is helpful in showing more significant deviations.

(10) $$\begin{array}{cc} \mathrm{RMSE} & =\sqrt{\frac{1}{N}\sum_{i=1}^{N}{\left(y_{i}-{\hat{y}}_{i}\right)}^{2}} \end{array}$$

(11) $$\begin{array}{cc} \mathrm{MAE} & =\frac{1}{N}\sum_{i=1}^{N}\left|y_{i}-{\hat{y}}_{i}\right| \end{array}$$

(12) $$\begin{array}{cc} \mathrm{MAPE} & =\frac{1}{N}\sum_{i=1}^{N}\left|\frac{y_{i}-{\hat{y}}_{i}}{y_{i}}\right|\times 100 \end{array}$$

where $y_{i}$ is the actual value and ${\hat{y}}_{i}$ is the prediction derived from the forecast model and *N* is the number of predictions of air pollution concentrations.

### 4.2. Predictionwith Hybrid Method SSA+LSTM

The performances of the hybrid approach and those used as reference are summarized in Table 1. As can be seen, the ${\mathrm{SSA}}_{\mathrm{signal}}$+${\mathrm{LSTM}}_{\mathrm{noise}}$ method achieved better results compared with the other methods. This highlights the importance of considering the integration of residual modeling using the LSTM network when forecasting PM_10_ concentrations. With a larger prediction amplitude, the errors in all the models increased; however, this was at a considerably slower rate for the predictive method. This reveals the prediction capacity of the hybrid method in different periods (short, medium, and long).

**Figure 14:**
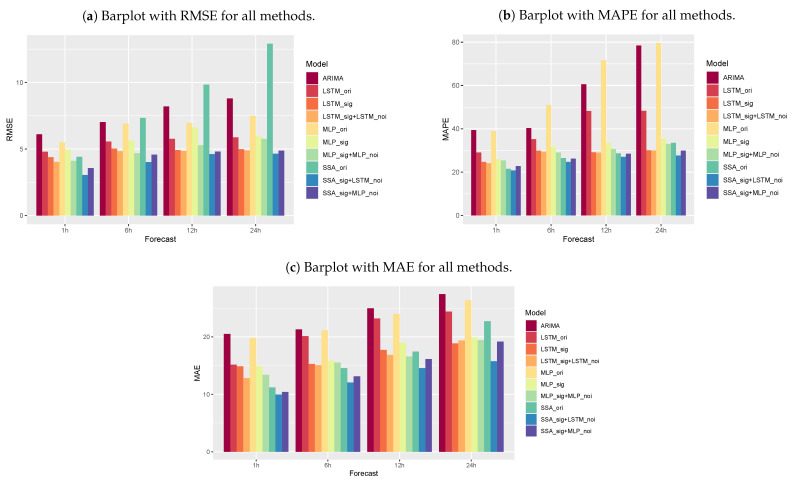
Accuracy measures (**a**) RMSE, (**b**) MAPE, and (**c**) MAE for all methods.

To further verify the effectiveness of the SSA+LSTM forecasting method, several popular air pollution forecasting methods, such as the MLP, LSTM, and MPL with signal and noise, and the LSTM with signal and noise, were considered for the forecasting performance comparison. Table 1 shows the RMSE, MAE, and MAPE of different forecasting methods (see also Figure 14a–c). It can be seen from Table 1 that (1) SSA+LSTM has the highest forecasting accuracy than other methods. (2) MLP and MLP with signal and noise have larger RMSE, MAE, and RMSE values in both one-step ahead and multi-step ahead predictions. (3) The proximity of the RMSE, MAE, and RMSE values for the method from the forecast for 6 h ahead is due to one of the properties of the LSTM network related to data memory; that is, it can learn long-term sequences of PM_10_ concentrations.

**Figure 15:**
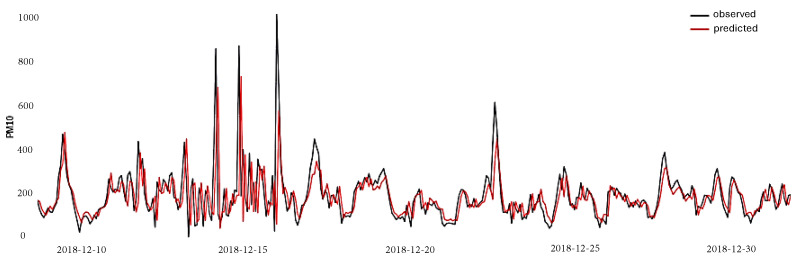
Behavior of the hybrid model between the observed PM_10_ values (represented by the black lines) and the predictions’ generated red lines).

Figure 15 shows the behavior of the hybrid method. The relationship between the observed PM_10_ values (represented by the black lines) and the predictions generated by the proposed hybrid method is illustrated, which offered, according to the performance metrics, the best performance. This proposed method consistently captures the general trends of the time series, reflecting the ability of the approach to model both seasonal patterns and fluctuations. Although some deviations could be observed at specific points, the proximity between the lines indicates a high prediction accuracy, highlighting the effectiveness of the SSA+LSTM method in addressing the inherent complexity of PM_10_ ambient pollution data. This performance suggests that the model is robust in accurately predicting PM_10_ concentrations, which can be crucial for environmental monitoring and management.

## 5. Conclusions and Future Scope

In this study, ten competing methods are presented. Seven of these adopt spectrum analysis components for air pollution forecasting for 1 h, 6 h, 12 h, and 24 h ahead. The methods were evaluated with the PM_10_ hourly time series between 2017 and 2019. The SSA preprocesses the data to extract the time series trend, seasonal, and noise components. Once the components were extracted, they were predicted with seven methods: multilayer perceptron with signal, multilayer perceptron with signal + multilayer perceptron with noise, long short-term memory with signal, long short-term memory with signal + long short-term memory with noise, singular spectrum analysis (signal only), singular spectrum analysis with signal + multilayer perceptron with noise, and finally, singular spectrum analysis with signal + long short-term memory with noise.

The hybrid method showed high precision in predicting PM_10_ and superiority concerning the individual methods (without preprocessing). Likewise, this method derived some variations based on SSA, which were superior to the rest of the methods presented with a conventional window length. The high gain achieved with this hybrid method lies in the SSA, which deals with the parts of trend and seasonality (linear part), and the LSTM network takes care of the nonlinear part (noise), having as its main virtue long-term dependence. This is observed not only an hour ahead but also six, twelve, and twenty-four hours ahead of the forecast. The evaluation through the performance metrics confirms the superiority of the hybrid method singular spectrum analysis plus long short-term memory over the individual methods.

The increasing availability of historical data and computational resources has facilitated the development of better-performing methods for air pollution prediction. In this study, a hybrid method based on nonparametric statistics and deep artificial neural networks for air pollution concentration forecasting was developed. This method is based on an integration of singular spectrum analysis and long short-term memory neural networks to take into account the modeling of the residuals extracted from its matrix decomposition and to evaluate it by adhering to the signal component in the prediction model. The hybrid method was first trained to confirm network parameters and then justified by verification with test sample data. The experimental results showed that the predictive method outperformed the other methods in predicting PM_10_ values. The performance advantage of the method is consistent for both the prediction and the error minimum fluctuation trend. With RMSE, MAE, and MAPE in the prediction of 1 h, 6 h, 12 h, and 24 h, it is verified that the hybrid method is an effective statistical method to estimate the concentration of PM_10_. Due to the promising results, this method will be extrapolated and evaluated with other time series related to air pollution and other areas of knowledge. As future research complements this developed predictive approach, addressing classification approaches and simultaneously developing other hybrid classification methods for air pollution concentrations have been considered. We can also develop a proposal that includes spatial covariates in the hybrid method and likewise create prediction intervals for all the methods addressed in this investigation. Finally, using the multivariate SSA followed by the LSTM, we can consider multivariate series. Indeed, there is a multivariate version of the singular spectrum analysis (multivariate SSA) that can be used in the first part of the hybrid methodology. However, its usefulness, compared with the univariate SSA, is highly dependent on the “correlation/relation” between the variables [47]. The assessment of this “correlation/relation” can be complex to find as we are dealing with time series, and measures such as dynamic time warping are more appropriate than the standard Pearson correlation. In future work, we will work on a deep study where these correlations between time series are considered and incorporated into the multivariate SSA to improve the results in a multivariate hybrid methodology. The potential of the SSA+LSTM model to adapt to different regions and pollutants positions it as a promising solution for air quality management and monitoring, and can also be extended to other scenarios with different datasets [62,63,64].

## Figures and Tables

**Table 1:** RMSE, MAE, and MAPE for all considered methods for 1 h, 6 h, 12 h, and 24 h ahead of out-of-sample forecasts. These values are based on a 10-fold cross-validation experiment.

Time	Method	RMSE	MAE	MAPE
+1h	ARIMA	6.10	20.50	39.43
	${\mathrm{MLP}}_{\mathrm{orig}}$	5.50	19.81	38.90
	${\mathrm{MLP}}_{\mathrm{sig}}$	4.97	14.93	25.95
	${\mathrm{MLP}}_{\mathrm{sig}}$+${\mathrm{MLP}}_{\mathrm{noi}}$	4.11	13.40	25.40
	${\mathrm{LSTM}}_{\mathrm{orig}}$	4.79	15.17	29.05
	${\mathrm{LSTM}}_{\mathrm{sig}}$	4.38	14.88	24.70
	${\mathrm{LSTM}}_{\mathrm{sig}}$+${\mathrm{LSTM}}_{\mathrm{noi}}$	4.03	12.84	24.09
	SSA	4.41	11.21	21.52
	${\mathrm{SSA}}_{\mathrm{sig}}$+${\mathrm{MLP}}_{\mathrm{noi}}$	3.56	10.42	22.74
	${\mathrm{SSA}}_{\mathrm{sig}}$+${\mathrm{LSTM}}_{\mathrm{noi}}$	3.04	9.98	20.74
+6h	ARIMA	7.02	21.3	40.32
	${\mathrm{MLP}}_{\mathrm{orig}}$	6.91	21.15	50.99
	${\mathrm{MLP}}_{\mathrm{sig}}$	5.63	15.89	31.71
	${\mathrm{MLP}}_{\mathrm{sig}}$+${\mathrm{MLP}}_{\mathrm{noi}}$	4.69	15.55	29.11
	${\mathrm{LSTM}}_{\mathrm{orig}}$	5.56	20.12	35.18
	${\mathrm{LSTM}}_{\mathrm{sig}}$	5.02	15.29	29.89
	${\mathrm{LSTM}}_{\mathrm{sig}}$+${\mathrm{LSTM}}_{\mathrm{noi}}$	4.83	15.07	29.44
	SSA	7.33	14.57	26.48
	${\mathrm{SSA}}_{\mathrm{sig}}$+${\mathrm{MLP}}_{\mathrm{noi}}$	4.58	13.14	26.21
	${\mathrm{SSA}}_{\mathrm{sig}}$+${\mathrm{LSTM}}_{\mathrm{noi}}$	4.02	12.07	24.69
+12h	ARIMA	8.20	24.97	60.58
	${\mathrm{MLP}}_{\mathrm{orig}}$	6.97	23.99	71.72
	${\mathrm{MLP}}_{\mathrm{sig}}$	6.63	18.99	33.55
	${\mathrm{MLP}}_{\mathrm{sig}}$+${\mathrm{MLP}}_{\mathrm{noi}}$	5.28	16.59	30.68
	${\mathrm{LSTM}}_{\mathrm{orig}}$	5.77	23.21	48.19
	${\mathrm{LSTM}}_{\mathrm{sig}}$	4.92	17.74	29.13
	${\mathrm{LSTM}}_{\mathrm{sig}}$+${\mathrm{LSTM}}_{\mathrm{noi}}$	4.85	16.88	29.02
	SSA	9.84	17.47	28.67
	${\mathrm{SSA}}_{\mathrm{sig}}$+${\mathrm{MLP}}_{\mathrm{noi}}$	4.81	16.17	28.51
	${\mathrm{SSA}}_{\mathrm{sig}}$+${\mathrm{LSTM}}_{\mathrm{noi}}$	4.62	14.57	27.05
+24h	ARIMA	8.80	27.45	78.43
	${\mathrm{MLP}}_{\mathrm{orig}}$	7.49	26.38	79.57
	${\mathrm{MLP}}_{\mathrm{sig}}$	5.97	19.89	35.37
	${\mathrm{MLP}}_{\mathrm{sig}}$+${\mathrm{MLP}}_{\mathrm{noi}}$	5.77	19.44	33.02
	${\mathrm{LSTM}}_{\mathrm{orig}}$	5.88	24.39	48.31
	${\mathrm{LSTM}}_{\mathrm{sig}}$	4.99	18.84	30.12
	${\mathrm{LSTM}}_{\mathrm{sig}}$+${\mathrm{LSTM}}_{\mathrm{noi}}$	4.89	19.37	30.02
	SSA	12.91	22.74	33.49
	${\mathrm{SSA}}_{\mathrm{sig}}$+${\mathrm{MLP}}_{\mathrm{noi}}$	4.87	19.16	29.94
	${\mathrm{SSA}}_{\mathrm{sig}}$+${\mathrm{LSTM}}_{\mathrm{noi}}$	4.65	15.79	27.62

## Data Availability

The datasets are available in the repository https://www.senamhi.gob.pe/site/descarga-datos/, accessed on 15 January 2023.
